# Sonodynamic and magnetic targeting platelet-membrane biomimetic platform for glioblastoma therapy

**DOI:** 10.3389/fbioe.2025.1648167

**Published:** 2025-09-23

**Authors:** Shibin Zou, Yueyang Zhang, Shanyong Mao, Mengxia Yuan, Jie Yang

**Affiliations:** ^1^ Department of Ultrasound Imaging, Chengdu Second People’s Hospital, Chengdu, China; ^2^ Department of Clinical Laboratory, Public Health Clinical Center of Chengdu, Chengdu, China; ^3^ Department of Functional Examination, Sichuan Provincial Orthopedic Hospital, Chengdu, China; ^4^ Department of Clinical Laboratory, Sichuan Clinical Research Center for Cancer, Sichuan Cancer Hospital & Institute, Sichuan Cancer Center, University of Electronic Science and Technology of China, Chengdu, China

**Keywords:** focused ultrasound, platelet, microbubble, magnetic targeting, glioblastoma

## Abstract

**Background:**

Due to the presence of the blood-brain barrier, the efficacy of chemotherapy for glioblastoma has remained suboptimal. Even drugs capable of crossing the BBB, such as temozolomide, exhibit limited therapeutic outcomes owing to insufficient targeting.

**Methods:**

Here, we developed a platelet membrane-hybridized biomimetic microbubble by loading superparamagnetic iron oxide and temozolomide-embedded polymers, constructing an ultrasound-controlled platelet membrane-inspired magnetic targeting drug delivery platform (ST-PM). Extensive *in vitro* and *in vivo* experimental results demonstrate that ST-PM exhibits inhibitory effects on glioblastoma under the combined action of a magnetic field and focused ultrasound.

**Results:**

The results demonstrated that ST-PM significantly enhanced microbubble stability *in vivo* while preserving both the magnetic targeting capability of magnetic drugs and the ultrasound-sensitizing properties of microbubbles. In orthotopic tumor mouse models, ST-PM exhibited superior tumor-targeting efficiency and markedly inhibited tumor growth. Furthermore, H&E staining of major organs confirmed the biosafety of ST-PM.

**Conclusion:**

The platelet membrane enhances the stability of drug-loaded microbubbles, facilitating targeted drug accumulation at the lesion site. These findings propose a novel treatment strategy with clinical translation potential for glioblastoma management.

## 1 Introduction

Glioblastoma (GBM) is the most common and aggressive primary brain tumor, characterized by high incidence, mortality, and recurrence rates (Weller et al., 2024). Despite advances in the development of novel therapeutics, the five-year survival rate for GBM has remained largely unchanged over the past decade (Corso and Bindra, 2016). One key reason is the presence of the blood-brain barrier (BBB), which prevents most drugs from reaching tumor sites (Pardridge, 2007). Even those capable of crossing the BBB, such as temozolomide (TMZ), face limitations due to insufficient targeting efficacy (Haar et al., 2012). Thus, the pursuit of innovative therapeutic modalities and strategies is clinically imperative to enhance survival and prognosis in GBM patients.

Focused ultrasound (FUS) holds great promise for the safe and effective opening of the BBB. The combination of microbubbles (MBs) with FUS represents an emerging technology that enables localized, noninvasive, and transient BBB opening, thereby enhancing drug delivery for brain tumors and other central nervous system disorders (Hynynen et al., 2001). The mechano-acoustic interplay of ultrasound, MBs, and microvasculature may trigger transient tight junction dissociation in lesion areas, facilitating active transport mechanisms and establishing a transient drug delivery window (Park et al., 2012; Park et al., 2017). *In vitro* studies have demonstrated that FUS combined with MBs can effectively enhance vascular endothelial gap formation and disrupt tight junctions between cells, thereby improving the penetration of biomolecules and drugs across the BBB and increasing paracellular flux between adjacent endothelial monolayers (Liu et al., 2014; Deng, 2010).

Although targeted BBB opening has achieved success, several challenges remain to be addressed for optimizing GBM therapeutic efficacy. Industrially manufactured lipid MBs exhibit poor biocompatibility and are prone to recognition by the host immune surveillance system during circulation, resulting in significant off-target depletion and potential immune rejection reactions, which substantially limit their clinical application (Qiao et al., 2021). The engineered cell membrane encapsulation strategy effectively enhances the stability, targeting ability and biological safety of drugs as well as various nanomaterials in the body (Ma et al., 2024). Among them, the platelet membrane, due to its ease of acquisition, high biocompatibility, and modifiability, has been widely studied in tumor-targeted drug delivery (Sun et al., 2019). Furthermore, drug delivery following BBB opening remains a passive process relying solely on free diffusion across the barrier. In contrast, magnetic nanoparticles possess physically responsive properties that enable reaction to external magnetic fields (Lammers et al., 2015). Magnetic targeting (MT) enables active enhancement of drug deposition at the target site, resulting in increased therapeutic drug concentrations—an outcome unattainable via passive diffusion (Fan et al., 2013). MT has been proven to play a significant role in the delivery system of nanoparticles for brain diseases, especially for brain tumors (Zhou et al., 2025; He et al., 2023).

**SCHEME 1:**
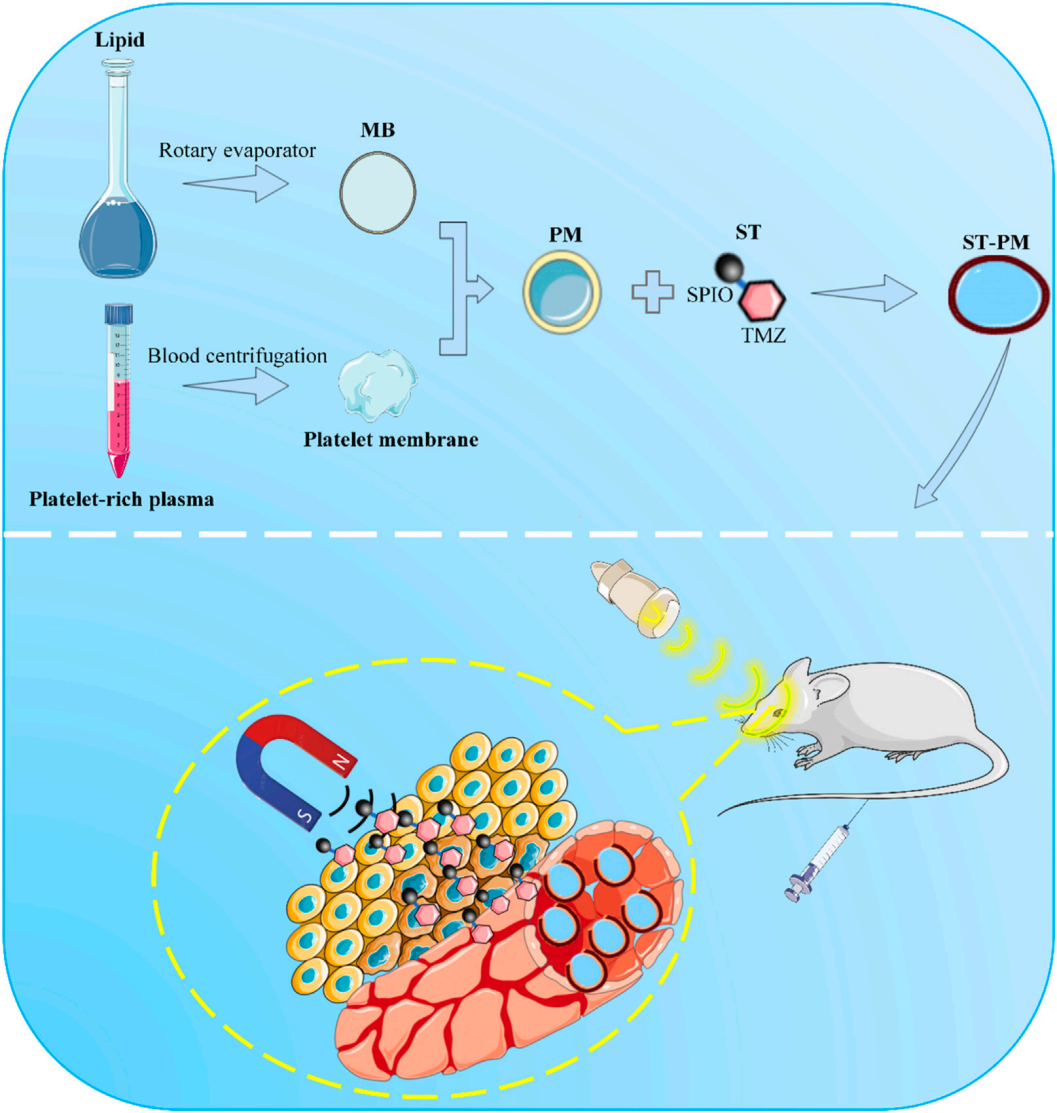
Schematic illustration of ST-PM penetrating the BBB for targeted treatment of GBM under the combined effect of FUS and MT.

Herein, we present a biomimetic microbubble hybridized with platelet membranes, which is engineered by loading a composite of SPIO nanoparticles and TMZ (SPIO-TMZ; ST), thereby constructing an ultrasound-responsive platelet-mimicking platform (ST-PM). The ST-PM exhibits prolonged *in vivo* stability, enabling it to synergistically achieve FUS-induced BBB opening and MT-guided drug delivery (Scheme 1). This platform actively targets GBM tissues, facilitating enhanced drug penetration into GBM regions upon FUS irradiation. Compared to conventional TMZ formulations, the ST-PM demonstrates superior therapeutic efficacy against GBM.

## 2 Materials and methods

### 2.1 Cell culture

The glioblastoma cell line GL261 was obtained from the American Type Culture Collection (ATCC, Manassas, VA, United States). Cells were cultured in Dulbecco’s Modified Eagle Medium supplemented with 10% fetal bovine serum and 1% penicillin-streptomycin, and maintained at 37 °C in a humidified 5% CO_2_ incubator.

### 2.2 Extraction of platelets and membranes

Mouse blood was collected in EDTA tubes and centrifuged at 200 *g* for 20 min at room temperature to obtain the supernatant containing platelet-rich plasma. Subsequently, a 5 mM prostaglandin E1 (PGE1) solution was mixed with phosphate-buffered saline (PBS) and added to the purified platelet concentrate. The mixture was then centrifuged at 1800 *g* for 20 min at room temperature to isolate the platelets. After removing the supernatant, the pelleted platelets were resuspended in PBS containing 5 mM PGE1 and subjected to multiple freeze-thaw cycles, followed by centrifugation at 8000 *g* for 15 min and sonication in a water-bath ultrasonicator for 2 min. Finally, the platelet membrane was aliquoted into 1 mL samples and stored at −80 °C until use.

### 2.3 Fabrication of SPIO-TMZ complex (ST)

The SPIO nanoparticles (purchased from Xi’an Delta Biological Technology Co., Ltd.) were dissolved in deionized distilled water to achieve a final concentration of 2 mg/ml. A TMZ solution (purchased from Selleck Chemicals LLC) was then added, and the mixture was homogenized using an ultrasonic agitator. The SPIO nanoparticles and TMZ were allowed to react naturally in a water bath at 37 °C for 4 h. Subsequently, PBS was slowly added, followed by magnetic precipitation to collect the ST complex. The collected ST solution was centrifuged at 11,000 × g for 3 min to separate the well-coupled ST composites from unbound TMZ. Finally, the complexes were resuspended in PBS.

### 2.4 Preparation of PM and ST-PM

Lipid mixtures consisting of 1,2-distearoyl-sn-glycero-3-phosphocholine (DSPC), 1,2-distearoyl-sn-glycero-3-phosphoethanolamine (DSPE), and distearoyl phosphoethanolamine-PEG2000 (DSPE-PEG2000) at a molar ratio of 21:21:1 (total 22 mg, all purchased from Xi’an Delta Biological Technology Co., Ltd.) were precisely weighed and transferred into glass vials. In a fume hood, 1 mL of organic solvent was added to each vial followed by vortex mixing until complete dissolution. The solutions were then incubated in a 65 °C water bath for 1 h to allow complete solvent evaporation, followed by an additional 20–30 min of heating. Subsequently, the samples were placed in a rotary evaporator for 8–12 h under vacuum to remove residual moisture. The vials were cooled at 4 °C for 1 h.

A PBS-glycerol buffer was prepared by adding 100 μL glycerol to 20 mL PBS and vortexing for 1 min before use. Then, 500–800 μL of the PBS-glycerol buffer was added to each vial, followed by incubation in a 65 °C water bath for 10 min. The mixtures were sonicated for 10 min, heated again at 65 °C for 3 min, and then supplemented with 4 mg of ST complex. If the extracted platelet membrane was added here before the ST complex was added, bionic microbubbles with platelet membrane hybridization (PM) were obtained. Confocal microscopy was used to observe the cell membranes and lipids stained by two kinds of fluorescent dyes respectively to confirm the successful hybridization of the two.

The vials were evacuated using an automatic vacuum pump and backfilled with C_3_F_8_ gas for 10 s. ST-PM were formed by mechanical agitation using a vial mixer for 45 s. The resulting ST-PM were stabilized by incubation at 4 °C for 30 min before subsequent experiments.

### 2.5 Transmission electron microscopy (TEM)

An appropriate aliquot of the prepared ST complex and ST-PM samples was deposited onto carbon-coated copper grids. The samples were allowed to air-dry completely before being mounted onto the TEM specimen holder. The focus was adjusted using the fine-focus knobs to locate suitable viewing areas. Once clear visualization was achieved, the size and morphology of the samples were observed and analyzed, and images were captured for documentation.

### 2.6 Dynamic light scattering (DLS)

The particle size and zeta potential of ST and ST-PM were analyzed using DLS with a Zetasizer Nano ZS90 instrument and its accompanying software (ZetaView 8.05.14). Samples were diluted in PBS prior to measurements of particle size, concentration, and membrane potential. All measurements were recorded and analyzed under controlled conditions, with temperature maintained at 25 °C and pH stabilized at 7.0.

### 2.7 The ST complex conjugation efficiency

The supernatant containing free TMZ was collected by centrifugation, while the pellet was resuspended in PBS to obtain the ST complex. Both free TMZ and conjugated ST complexes were nitrated and quantified using inductively coupled plasma atomic emission spectroscopy (ICP-AES). The conjugation efficiency was calculated using the following formula:

$$\text{Conjugation efficiency }\left(\%\right)=\frac{Wconjugated\;ST}{Wconjugated\;ST+Wfree\;TMZ}\times 100\%$$

W_conjugated ST_ means the amount of conjugated ST, and W_free TMZ_ means the amount of free TMZ.

### 2.8 Magnetic targeting and ultrasound imaging *in vitro*

A 0.48 T permanent magnet was applied to the ST-PM solution for 2 min, with solution changes documented photographically. For ultrasound imaging, a 2% agarose phantom was prepared by dissolving 2 g agarose in 100 mL 1×TAE buffer (microwaved for 5 min until clear), cast into a mold, and embedded with three 1.0 cm-diameter wells created using 1.5 mL EP tubes. After solidification, 500 μL of 10×PBS-diluted ST-PM solution was loaded into the wells and imaged using a clinical ultrasound system (10 MHz linear transducer), with probe alignment ensuring simultaneous visualization of all wells and images acquired at 10-min intervals.

### 2.9 Confocal laser scanning microscope (CLSM)

After establishing an *in vitro* BBB model, GL261 cells were seeded in the lower chamber with glass-bottom wells. After 24 h of culture, fresh serum-free medium containing ST-PM (note: since TMZ lacks fluorescence, this study substituted it with Cy3 and SPIO conjugates, collectively referred to as ST complexes for presentation purposes) was added to the dosing chamber. The medium was then exposed to a 0.48 T permanent magnet with or without MT for 10 min, followed by 5 min of FUS treatment (or no treatment, depending on the experimental group). Following 6 h of incubation, the lower chamber was thoroughly washed with PBS. Cells were fixed with 4% paraformaldehyde at room temperature for 20 min, then stained with 500 μL of 500 nmol/L DAPI solution for nuclear visualization (15 min). Finally, intracellular accumulation of the complexes in GL261 cells was examined using confocal laser scanning microscopy.

### 2.10 Cell counting kit-8 (CCK-8)

GL261 cells were seeded in 96-well plates at a density of 4 × 10^3^ cells per well. The cells were then co-incubated with PBS, TMZ, and ST-PM for 2 h under a 0.48 T permanent magnet, with or without MT. Subsequently, the cells were exposed to FUS for 5 min (or left untreated). After 48 h of incubation, 10 µL of CCK-8 solution was added to each well, followed by an additional 1-h incubation. Absorbance was then measured at 450 nm to assess cell viability.

### 2.11 Orthotopic GBM animal model

Following gas anesthesia, mice were secured in a stereotaxic frame for small animals. After shaving and disinfecting the surgical site, a midline scalp incision was made to expose the skull. The meninges were then treated with medical-grade hydrogen peroxide applied with a cotton swab. A small cranial burr hole (approximately 1.0 mm in diameter) was drilled using a dental drill at coordinates 1.0 mm anterior to bregma and 2.0 mm lateral to the sagittal suture. Subsequently, 2 × 10^5^ GL261 cells suspended in 10 μL PBS were slowly injected at a depth of 3.0 mm using a micro-syringe. The burr hole was sealed with bone wax, and the incision was closed with surgical sutures. Tumor growth was monitored by *in vivo* imaging system a week post-implantation, followed by experimental procedures according to the study protocol.

All animal experiments followed the guidelines for animal care and use of experimental animals published by the Institutional Animal Care and Use Committee of Sichuan Cancer Hospital (Grant No. SCCHEC-04-2020-004).

### 2.12 Magnetic and FUS experimental setup *in vivo*

After depilating the mouse head, the tumor location was identified using the imaging mode of the FUS system. Upon target confirmation, therapeutic FUS was delivered with the following parameters: sinusoidal waveform, PRF: 1100 kHz, amplitude: 1 V, and sonication duration: 4 min. Post-FUS treatment, a 0.48 T permanent magnet was securely fixed to the treated area, maintaining direct contact with the tumor site for 3 h before magnet removal for subsequent experiments.

### 2.13 Hematoxylin-eosin (H&E) staining

After baking the paraffin-embedded tissue sections at 60 °C for 1 h, dewax them sequentially in xylene I (15 min) and xylene II (15 min), then rehydrate through graded ethanol solutions (100%, 95%, 80%, and 70%, 5 min each). Stain with hematoxylin for 5 min, rinse with distilled water, differentiate in acid alcohol for 30 s, and wash twice in distilled water (5 min each). Counterstain with eosin for 1 min, remove excess dye, then rapidly dehydrate through ethanol series (70%, 80%, 95%, and 100%–2 s each), followed by a 1-min immersion in 100% ethanol. Clear the sections in xylene (twice, 1 min each), mount with neutral balsam, and examine under a microscope for imaging.

### 2.14 Statistical analysis

All data are presented as mean ± standard deviation. Intergroup comparisons were performed using two-tailed paired Student's t-tests for pairwise comparisons, while one-way analysis of variance followed by Tukey’s *post hoc* test was employed for multiple group comparisons. All statistical analyses were conducted using SPSS (version XX, IBM Corp., Armonk, NY, United States) and GraphPad Prism (version 6.0, GraphPad Software, San Diego, CA, United States). A p-value of <0.05 was considered statistically significant.

## 3 Results

### 3.1 Preparation, characterization and identification of ST-PM

**FIGURE 1:**
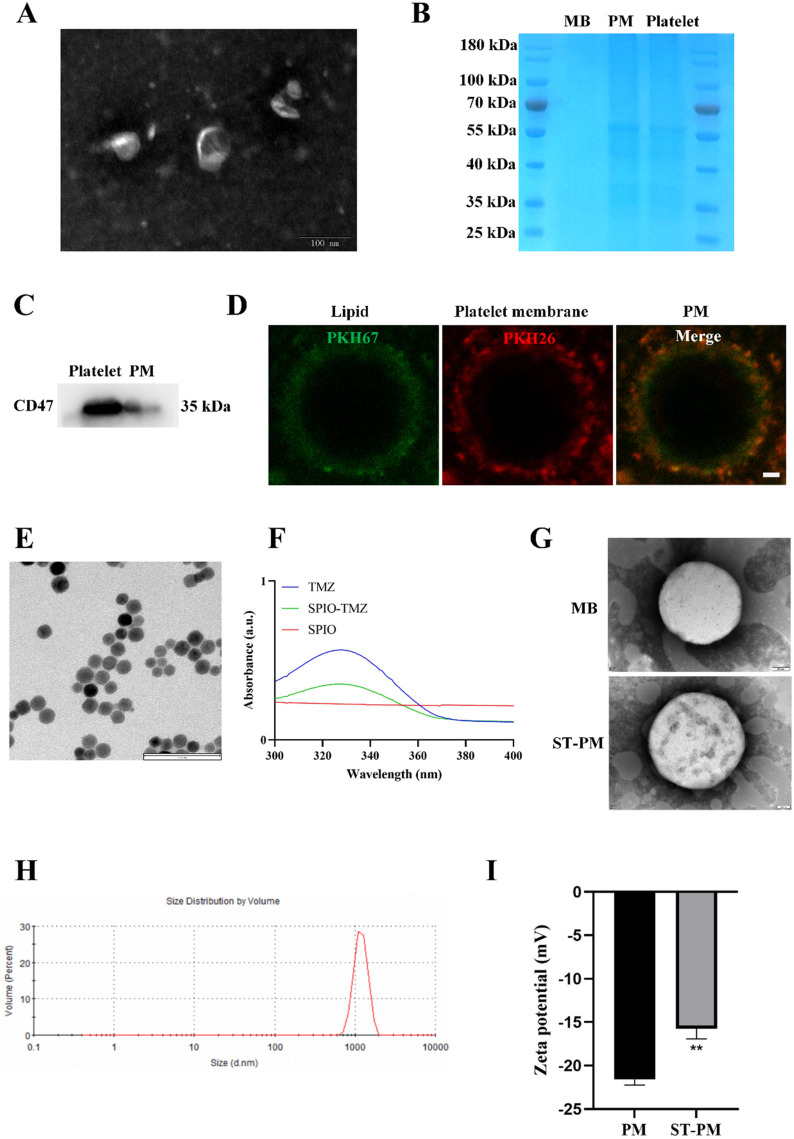
Characteristics of ST-PM. **(A)** TEM images of platelet membranes. **(B)** SDS-PAGE protein analysis of MBs, PM and platelets. **(C)** Western blot analysis was performed to measure protein expression levels of CD47. **(D)** Platelet membranes and lipid components with PKH67 and PKH26 fluorescent dyes. Scale bar = 1 μm. **(E)** TEM images of ST. **(F)** UV-VIS spectra of ST and its constituents. **(G)** TEM images of MBs and ST-PM. **(H)** Size distribution of ST-PM. **(I)** Zeta potential of ST-PM and PM. (n = 3, x ± SD. **P < 0.01).

We isolated the membrane from mouse whole blood-derived platelets, and TEM analysis revealed the characteristic bilayer membrane structure (Figure 1A). Subsequently, the platelet membrane was fused with lipid components to prepare PM, which exert inherent biological effects through membrane proteins derived from platelet membrane components. To verify the presence of membrane proteins, we performed protein gel electrophoresis staining assays comparing protein samples from platelet membranes, PM, and conventional lipid microbubbles. The gel electrophoresis results demonstrated nearly identical protein band patterns between PM and platelet membranes (Figure 1B). Furthermore, Western blot analysis confirmed the presence of the platelet-specific marker protein CD47 in PM (Figure 1C). To better visualize the fusion phenomenon between platelet membrane components and lipid constituents, we separately labeled platelet membranes and lipid components with PKH67 and PKH26 fluorescent dyes, respectively, to examine the structure of PM (Figure 1D). We conducted quantitative fluorescence co-localization analysis on this, and the results showed that the Pearson’s correlation coefficient was 0.48 (Supplementary Material 1). These results collectively demonstrate the successful preparation of PM with preserved platelet membrane proteins on their surface to ensure biological functionality.

We subsequently prepared the ST complex by incubating SPIO particles with TMZ in a water bath at 37 °C for 4 h, utilizing the natural reaction between amino and carbonyl groups. After purification to remove impurities, we conducted comprehensive characterization of the composite. TEM imaging revealed that the ST complex exhibited uniform spherical morphology with dark-colored particles due to their iron oxide content. Scale bar measurements indicated an average particle size of approximately 20 nm (Figure 1E). Subsequent UV-VIS spectroscopy analysis determined the TMZ loading efficiency to be 20.3% ± 1.6% (Figure 1F). The Fourier transform infrared spectroscopy results confirmed that the ST complex had the characteristic peaks of TMZ and SPIO (Supplementary Material 2A). These results collectively demonstrate the successful synthesis of a homogeneous ST nanocomposite with uniform particle morphology (∼20 nm) through the spontaneous reaction between SPIO particles and TMZ, achieving a therapeutic TMZ loading concentration.

Next, we encapsulated the ST complex within PM. To confirm successful encapsulation, we conducted a series of characterization experiments. TEM imaging revealed the morphological structure of the drug-loaded MBs. Compared to blank MBs, ST-PM exhibited greater internal turbidity, along with visible ST complexes on their surface (Figure 1G). DLS confirmed that ST-PM remained in the micrometer size range, meeting the criteria for ultrasound microbubbles (Figure 1H). Furthermore, zeta potential measurements verified the effective loading of the ST into PM. The zeta potentials of blank PM and ST-PM were −21.59 ± 0.53 mV and −15.76 ± 0.95 mV, respectively, demonstrating successful encapsulation (Figure 1I). In summary, these results confirm the successful synthesis of both the ST complex and PM, as well as their effective loading. Through comprehensive characterization—including size, morphology, and surface potential analysis—we conclusively validated the successful preparation of ST-PM.

### 3.2 Functional characteristics of ST-PM *in vitro*

**FIGURE 2:**
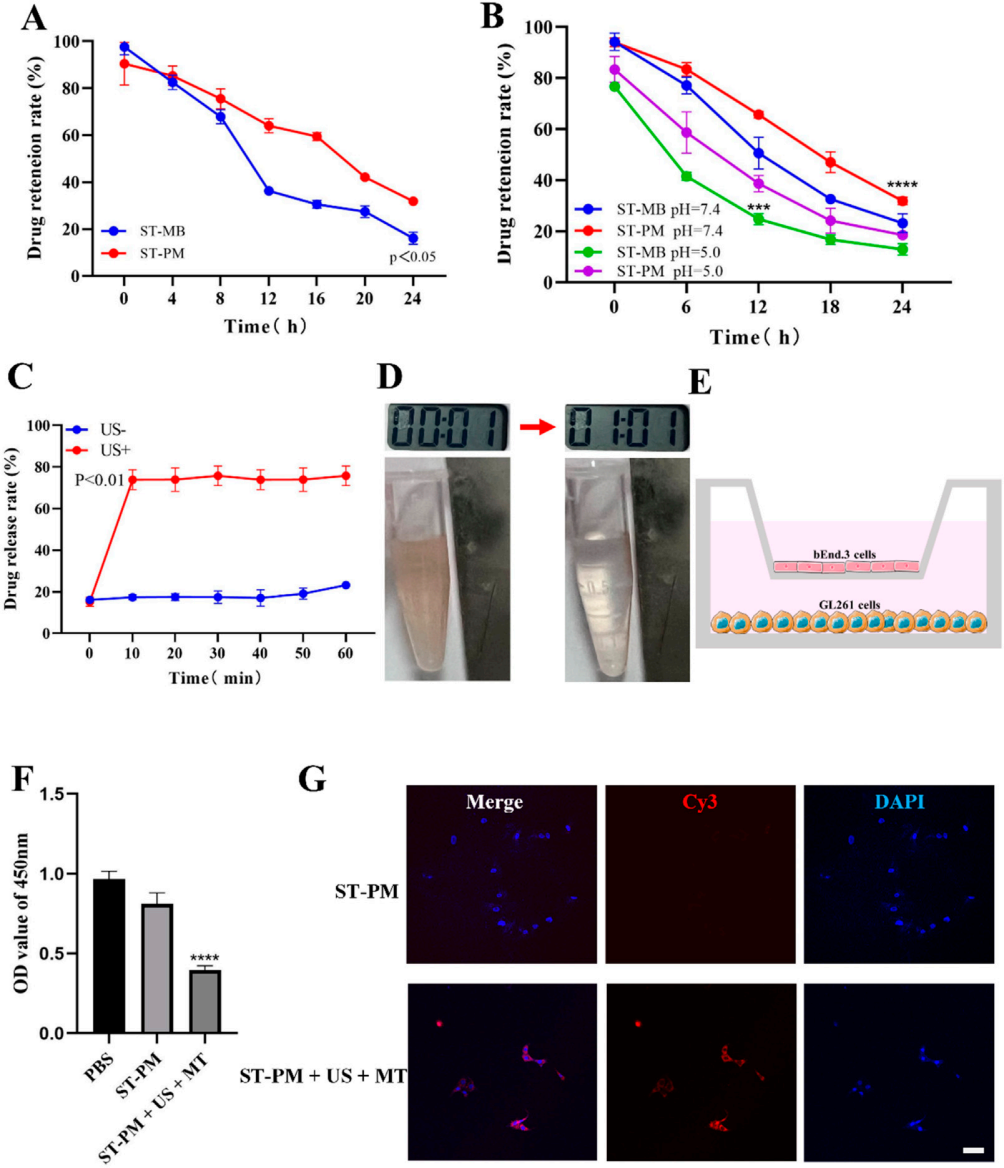
*In vitro* experimental results of ST-PM. **(A)** Serum stability of ST-MB and ST-PM. **(B)** Different pH levels stability of ST-MB and ST-PM. **(C)** Drug release of ST-PM after ultrasonic irradiation. **(D)** Saturation magnetization of ST-PM. **(E)** Schematic illustrations of *in vitro* BBB model. **(F)** Cell viability after treatment. **(G)** The accumulation of ST-PM in GL261 cells. Scale bar = 50 μm. (n = 3, x ± SD. ****P < 0.0001).

We conducted a series of *in vitro* functional assays to systematically validate the functionalities conferred by each component of ST-PM. Since PM is fundamentally microbubble-based, we first investigated whether PM retained the functional characteristics of microbubbles. Comparative analysis with clinical ultrasound contrast agents (Supplementary Material 2B) demonstrated that PM exhibited comparable imaging performance to SonoVue^®^, confirming that the platelet membrane hybridization did not compromise the inherent contrast-enhancing capability of microbubbles under ultrasonic excitation. Notably, different pH levels and serum stability experiments revealed that the platelet membrane endowed PM with significantly enhanced stability (Figures 2A,B), a critical feature for improving drug delivery efficiency and therapeutic outcomes. Subsequently, we verified the drug release effect of ST-PM under the influence of ultrasound. The results showed that after ultrasound irradiation at the 10th minute, ST-PM effectively achieved drug release (Figure 2C).

Furthermore, leveraging the intrinsic magnetic properties of SPIO, we verified the magnetization capacity of ST-PM through external magnetic field experiments. When exposed to a permanent magnet for 2 min, suspended ST-PM particles exhibited effective magnetic attraction (Figure 2D), demonstrating sufficient magnetization for potential *in vivo* magnetic targeting applications.

In summary, ST-PM successfully integrates the functional properties of both SPIO and microbubbles, exhibiting dual capabilities of magnetic targeting and ultrasound sensitivity. The platelet membrane coating further confers superior stability to the construct. These combined characteristics position ST-PM as a highly promising platform for targeted drug delivery.

### 3.3 *In vitro* BBB opening and anti-tumor effects of ST-PM

We established an *in vitro* BBB model by seeding sufficient brain microvascular endothelial cells in the upper chamber of Transwell plates and culturing them to optimal confluence (Figure 2E). Using this model, we evaluated the BBB penetration efficiency of ST-PM under combined FUS and magnetic field exposure, followed by assessments of cellular uptake and cytotoxicity. Our results demonstrated that ST-PM could effectively cross the *in vitro* BBB model following FUS irradiation. Notably, the synergistic application of FUS with magnetic field guidance significantly enhanced both BBB transmigration of ST-PM (Cy3) and their subsequent uptake by GL261 cells (Figure 2G). These *in vitro* findings confirm that ST-PM can efficiently penetrate the BBB under FUS exposure, with magnetic field application further improving tumor cell internalization, thereby providing a solid experimental foundation for subsequent *in vivo* studies.

Subsequently, we evaluated the cytotoxic effects of ST-PM on GBM cells under the combined application of FUS and magnetic fields. CCK-8 assay results demonstrated that ST-PM exhibited the highest efficacy in killing GL261 cells when both FUS and magnetic fields were applied, outperforming all other experimental groups (Figure 2F). This finding indicates that the synergistic action of ultrasound and magnetic fields promotes effective drug release from ST-PM *in vitro*, achieving a therapeutic concentration sufficient to induce tumor cell death. Taken together, these results confirm that the combined stimulation of FUS and magnetic fields maximizes the therapeutic potential of ST-PM, enabling targeted treatment of GBM.

### 3.4 FUS combined with magnetic field enables ST-PM to precisely target GBM *in vivo*

**FIGURE 3:**
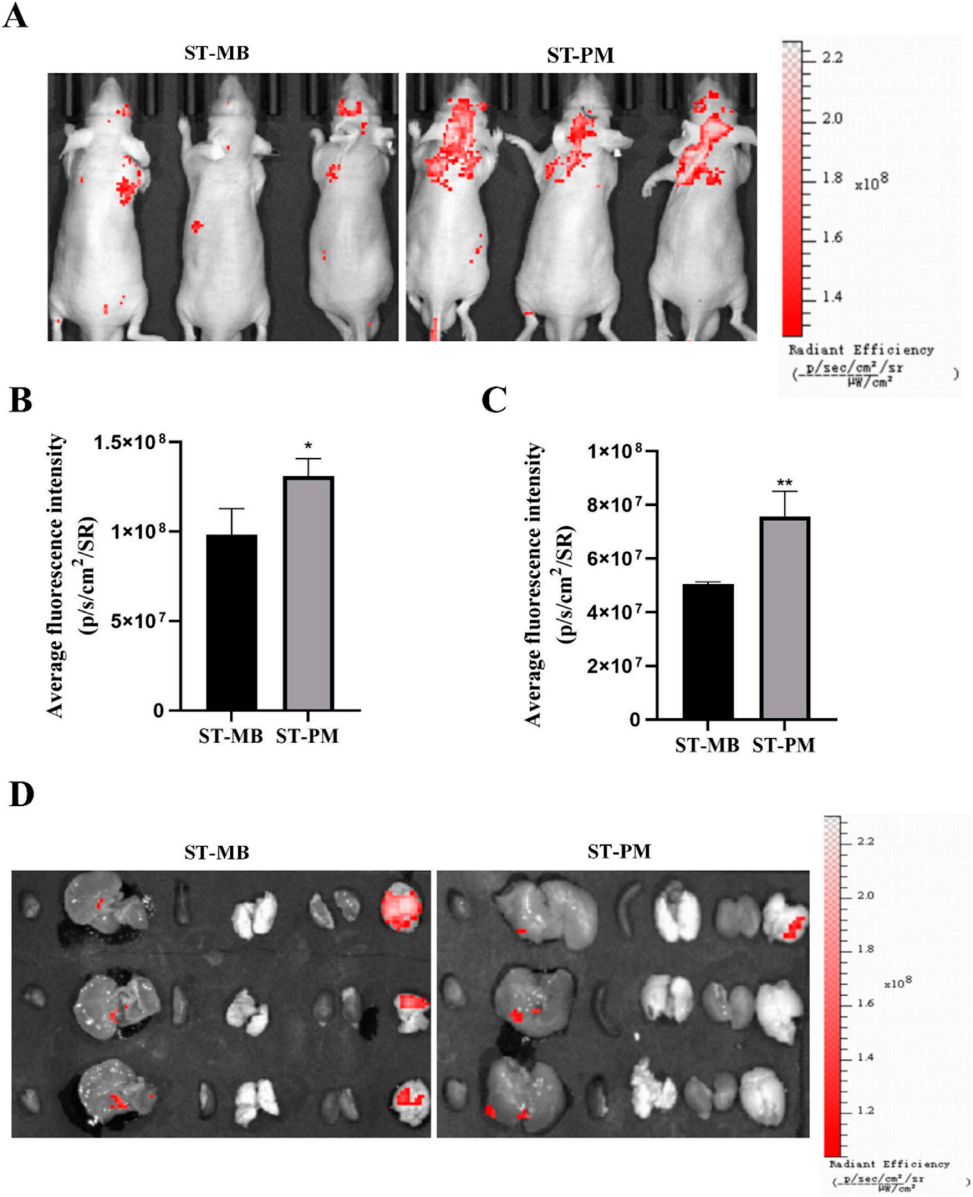
Biodistribution of ST-PM in orthotopic GBM mice. **(A)** Distributions of ST-MB and ST-PM visualized by IVIS Lumina II *in vivo* optical imaging. **(B)** The fluorescence intensity of ST-MB and ST-PM to brain in orthotopic GBM mice. **(C)** Fluorescence in intact organs in orthotopic GBM mice 12 h after intravenous injection of ST-MB and ST-PM. **(D)** Semi-quantitative statistic results of fluorescence intensity in main organs collected 12 h after injection of ST-MB and ST-PM. (n = 3, x ± SD. *P < 0.05, **P < 0.01).

We established an orthotopic GBM mouse model to evaluate the biodistribution of ST complexes and ST-PM (Cy3) following intravenous administration via the tail vein, with combined application of FUS and magnetic field. At 12 h post-injection of either ST complexes or ST-PM (Cy3) under the combined physical stimuli, the ST-PM (Cy3) group exhibited significantly stronger fluorescence intensity in the orthotopic GBM tumors compared to controls (Figures 3A,B), demonstrating enhanced ST accumulation *in vivo*. This result is consistent with the conclusion of the *in vitro* experiment, indicating that the platelet membrane effectively enhances the stability of ST-PM in the body.

Subsequently, at 12 h post-injection of either the ST complex or ST-PM (Cy3) combined with FUS and magnetic field exposure, the mice were euthanized for organ extraction, including the heart, liver, spleen, lungs, kidneys, and brain, to assess fluorescence distribution across different tissues at various time points (Figures 3C,D). The ST-PM (Cy3) group exhibited significantly higher accumulation in the brain compared to the ST complex alone, demonstrating that ST-PM, under the combined effects of FUS and magnetic fields, possess targeting capability toward brain tumors *in vivo*. Notably, due to the micrometer-scale size of the drug-loaded MBs, they are readily absorbed by the reticuloendothelial system during hepatic circulation, resulting in consistently strong fluorescence signals in the liver. This finding further confirms the efficient hepatic metabolism of the drug-loaded platform, supporting its *in vivo* safety profile.

In summary, under the synergistic application of FUS and magnetic fields, ST-PM successfully facilitated BBB opening, thereby enhancing drug accumulation in the brain and increasing drug concentration in the tumor region. It is worth noting that this result is consistent with the results obtained from *in vitro* cell uptake experiments. This approach holds significant potential for improving the therapeutic efficacy against GBM.

### 3.5 FUS combined with magnetic field enables ST-PM to effectively treat GBM

**FIGURE 4:**
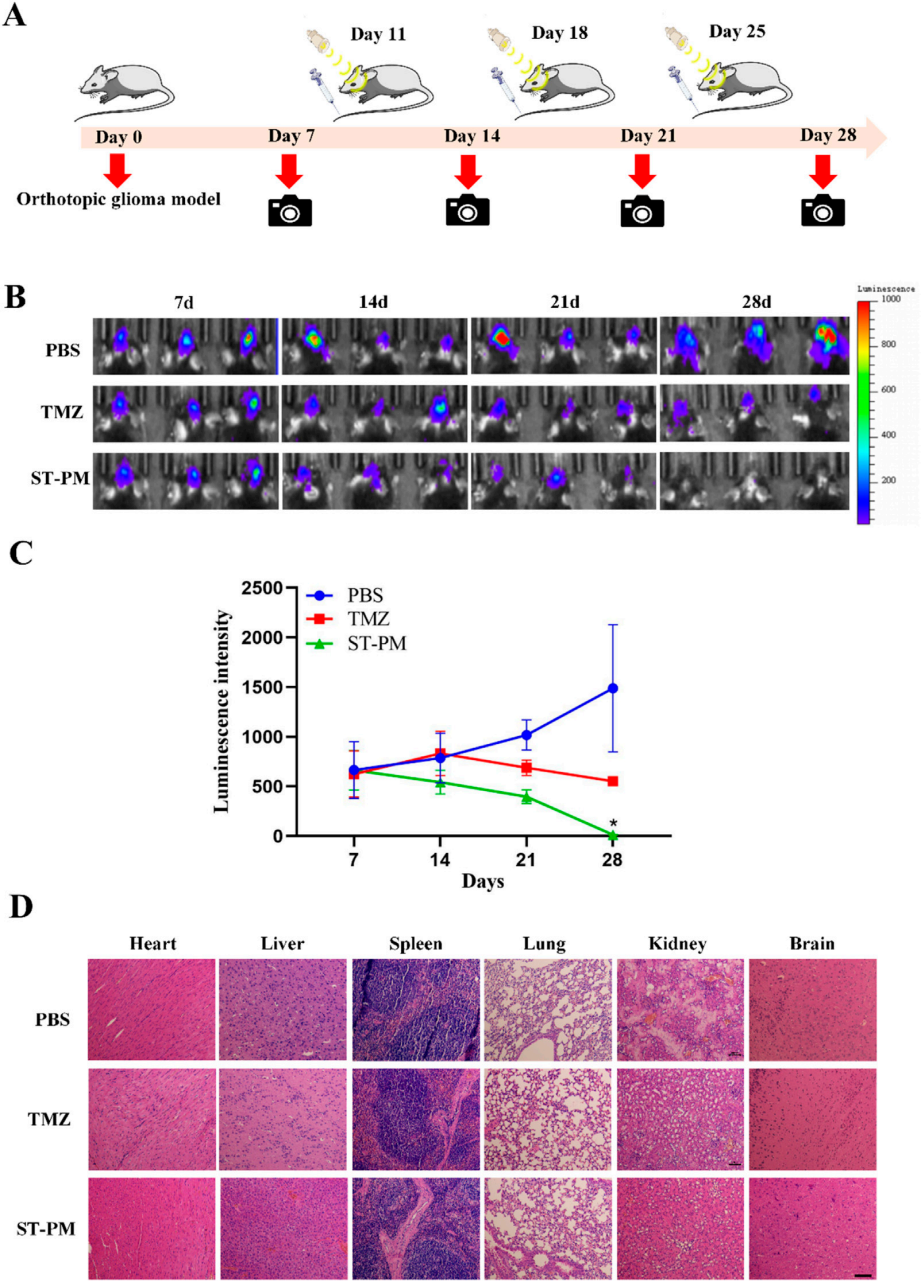
The therapeutic effect and biosecurity of ST-PM on orthotopic GBM mice. **(A)** Schematic diagram of treatment schedule. **(B)** GBM growth monitored by bioluminescence imaging. **(C)** Semiquantitative statistic results of luminescence intensity. **(D)** H&E staining of main organs of orthotopic GBM mice. (n = 3, x ± SD. *P < 0.05).

Using an orthotopic GBM mouse model, we evaluated the therapeutic efficacy of ST-PM against glioblastoma under combined FUS and magnetic field exposure. PBS, TMZ, or ST-PM were administered via tail vein injection followed by concurrent FUS and magnetic field application, with the PBS-injected group serving as the blank control. Treatments were performed twice weekly, and tumor progression was monitored using *in vivo* bioluminescence imaging. As the establishment of the orthotopic GBM mouse model requires approximately 1 week, baseline imaging was initiated on day 7 post-modeling, with comprehensive combination therapy beginning on day 9 and continuing until day 28 (Figure 4A).

Through longitudinal monitoring using *in vivo* bioluminescence imaging at each time point, we observed a significant increase in luminescence signals in the PBS control group after five treatment cycles, indicating continuous tumor growth. In contrast, both TMZ and ST-PM treatment groups showed attenuated bioluminescence signals in orthotopic GBM tissue, with the ST-PM group demonstrating more pronounced suppression (Figure 4B). Subsequent quantitative analysis of the tumor bioluminescence data recorded by the small animal imaging system revealed significantly reduced fluorescence intensity in the ST-PM treatment group compared to both PBS and TMZ groups (Figure 4C). These results demonstrate that ST-PM, under the combined effects of FUS and magnetic field, effectively inhibited GBM progression and significantly enhanced therapeutic outcomes. In conclusion, our findings indicate that the synergistic application of FUS and magnetic field with ST-PM markedly improves the therapeutic efficacy of TMZ against GBM compared to both PBS control and TMZ treatment alone.

### 3.6 Biological safety of ST-PM

At the conclusion of the 28-day treatment period, the mice were euthanized and dissected to harvest their major organs (heart, liver, spleen, lungs, kidneys, and brain) for evaluating the biosafety profile of ST-PM under combined FUS and magnetic field exposure in the orthotopic GBM mouse model. Histopathological examination via H&E staining revealed no significant pathological alterations in any of the collected organs from ST-PM-treated mice. The stained tissue sections showed no evidence of hemorrhage, inflammatory infiltration, or other morphological abnormalities across all examined organs (Figure 4D).

These findings demonstrate that ST-PM administration in conjunction with FUS and magnetic field application causes no detectable adverse effects in the animal model, indicating biosafety characteristics. This favorable safety profile underscores the translational potential of this therapeutic approach for clinical applications.

## 4 Discussion

GBM is the most common primary malignant brain tumor. The significant challenge in effectively treating GBM stems largely from the inability of many chemotherapeutic agents to penetrate the BBB. FUS combined with MBs, which enables localized and reversible BBB opening, has been extensively investigated for this purpose, a concept initially proposed as early as 2006 (Lentacker et al., 2006). Utilizing this approach for targeted chemotherapeutic drug delivery is considered a highly promising strategy with clinical potential for treating brain tumors. Drug-loaded MBs are currently widely explored in research for various cancer therapies (Li et al., 2022). To better leverage this promising platform, whole blood samples were collected from mice and platelet membranes were isolated. TEM imaging confirmed the presence of the characteristic bilayer membrane structure. Platelet membrane vesicles were then fused with lipid components to generate PMs hybridized nanoparticles. Gel electrophoresis with protein staining revealed that the protein bands of the PMs were nearly identical to those of the native platelet membranes. Furthermore, Western blot analysis confirmed the presence of the platelet-specific marker protein CD47 on the PMs. These results collectively demonstrate the successful preparation of platelet-membrane hybridized PMs, which retain key platelet membrane proteins essential for their intended biological functions. Subsequently, a magnetic drug complex ST was synthesized via the spontaneous reaction between the amino groups of SPIO nanoparticles and the carbonyl groups of TMZ. TEM characterization of the ST complex revealed a morphology with a diameter of approximately 20 nm. UV-Vis spectroscopy determined the TMZ loading capacity within the ST complex to be 20.3% ± 1.6%. The stable morphology, small size, and sufficient drug loading endow the ST complex with significant potential for GBM therapy. Finally, the ST complex was effectively encapsulated within the PMs to form the final construct, ST-PM.

The prepared ST-MBs exhibited no impairment of the inherent functionalities of their individual components. Their intrinsic capabilities, including magnetism and ultrasound contrast enhancement, were effectively preserved as demonstrated through a series of *in vitro* experiments. We initially confirmed that the PMs possess contrast functionality under ultrasound irradiation, indicating that platelet membrane hybridization did not compromise the core functions of the microbubbles. Notably, experiments conducted under varying pH conditions and serum stability assays revealed that the platelet membrane conferred enhanced stability upon the PMs. This enhanced stability is crucial for improving drug delivery efficiency and therapeutic efficacy. Furthermore, due to the inherent magnetism of SPIO, *in vitro* magnetic field manipulation experiments demonstrated that the ST-PMs exhibit high magnetizability. This property enables their potential for magnetic targeting *in vivo*. Collectively, these characteristics endow ST-PMs with significant promise for targeted drug delivery.

Based on these findings, we established an *in vitro* BBB co-culture model, which demonstrated that ST-PMs possess the capability to open the BBB under FUS exposure. Furthermore, following BBB penetration, the ST complex was effectively internalized by GBM cells under magnetic guidance, exerting its therapeutic effect against tumor cells. Subsequently, we administered ST-PMs via tail vein injection to orthotopic GBM-bearing mouse models to evaluate their biodistribution and anti-tumor efficacy under the combined application of FUS and magnetic fields. *In vivo* imaging performed 12 h post-injection revealed significantly enhanced *in vivo* stability of ST-PMs compared to conventional lipid microbubbles, consistent with the *in vitro* results. *Ex vivo* fluorescence imaging of dissected organs showed substantially higher accumulation of ST-PMs in the brain compared to the conventional microbubble group. These results elucidate that the combined FUS and magnetic field strategy significantly improves the systemic stability of ST-PMs and their accumulation in brain tissue. Finally, H&E staining of tissue sections from the brain, heart, liver, spleen, lungs, and kidneys confirmed the biosafety profile of ST-PMs. In summary, the synergistic application of FUS and magnetic fields with ST-PMs achieves BBB opening while enhancing therapeutic efficacy against glioma through improved targeting capability and favorable biosafety.

However, this study has several limitations. First, a key limitation is the lack of direct quantitative magnetic characterization data due to equipment constraints; future work utilizing relevant instrumentation will incorporate these measurements to further validate the findings. Second, regarding experimental design, critical *in vivo* studies such as biodistribution and tumor suppression were conducted with a sample size of n = 3 per group. While statistically significant results were achieved, satisfying the 4R principles (Reduction, Refinement, Replacement, Responsibility) advocated by animal protection organizations and welfare guidelines—specifically minimizing animal numbers while ensuring statistical validity (Kang et al., 2022)—this sample size remains statistically insufficient and represents an aspect requiring supplementation in future research. In subsequent studies, we will endeavor to overcome barriers to clinical translation, including the immunogenicity of murine-derived membranes, the heterogeneity of human GBM models, and the limitations of the GL261 cell line. Additionally, the molecular mechanisms underlying FUS-mediated microbubble cavitation for blood-brain barrier opening have prompted further investigation and will constitute a significant focus of our future research.

## 5 Conclusion

In this study, we developed platelet membrane-hybridized biomimetic microbubbles co-loaded with SPIO and TMZ. ST-PMs retained the inherent functional properties of each component while gaining enhanced stability from the platelet membrane coating, which is crucial for improving drug delivery efficiency and therapeutic outcomes. Under the combined action of focused ultrasound and magnetic fields, ST-PMs effectively opened the BBB and achieved precise, sufficient delivery of TMZ to GBM tumor tissues, thereby maximizing therapeutic efficacy. Our results demonstrate that ST-PMs, when combined with focused ultrasound and magnetic field guidance, possesses significant clinical translation potential as a promising therapeutic platform for GBM treatment.

## Data Availability

The raw data supporting the conclusions of this article will be made available by the authors, without undue reservation.
