# Supplementary material for: Sonodynamic and magnetic targeting platelet-membrane biomimetic platform for glioblastoma therapy
Source: Front Bioeng Biotechnol. 2025 Sep 23;13:1648167. doi: 10.3389/fbioe.2025.1648167 (PMC12500637; doi:10.3389/fbioe.2025.1648167)

Figure legends

A. Fourier transform infrared spectroscopy of TMZ, SPIO, and ST complex.

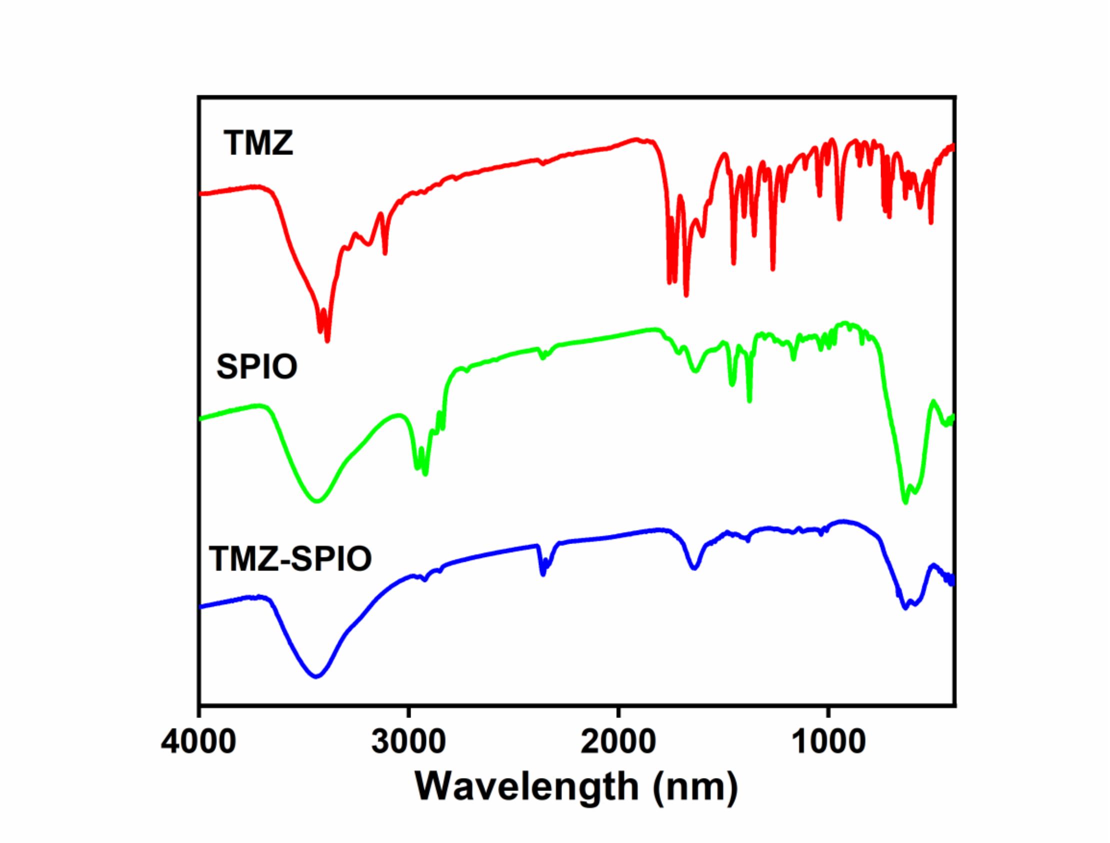

B. Ultrasound imaging of SonoVue, ST-PM, and double distilled water (DDW).

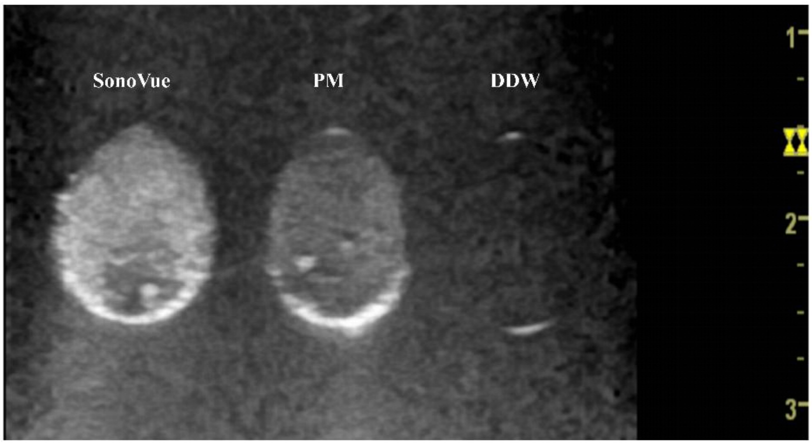

Supplement: Supplementary file 1 [file DataSheet2.pdf]
