# Supplementary material for: Sonodynamic and magnetic targeting platelet-membrane biomimetic platform for glioblastoma therapy
Source: Front Bioeng Biotechnol. 2025 Sep 23;13:1648167. doi: 10.3389/fbioe.2025.1648167 (PMC12500637; doi:10.3389/fbioe.2025.1648167)
Supplement: Supplementary file 2 [file DataSheet1.pdf]

Colocalization_of_Result of 1D1.tif (red)_versus_Result of 1D1.tif (green)_223345527.pdf

Li - Ch1

256.0 x 256.0

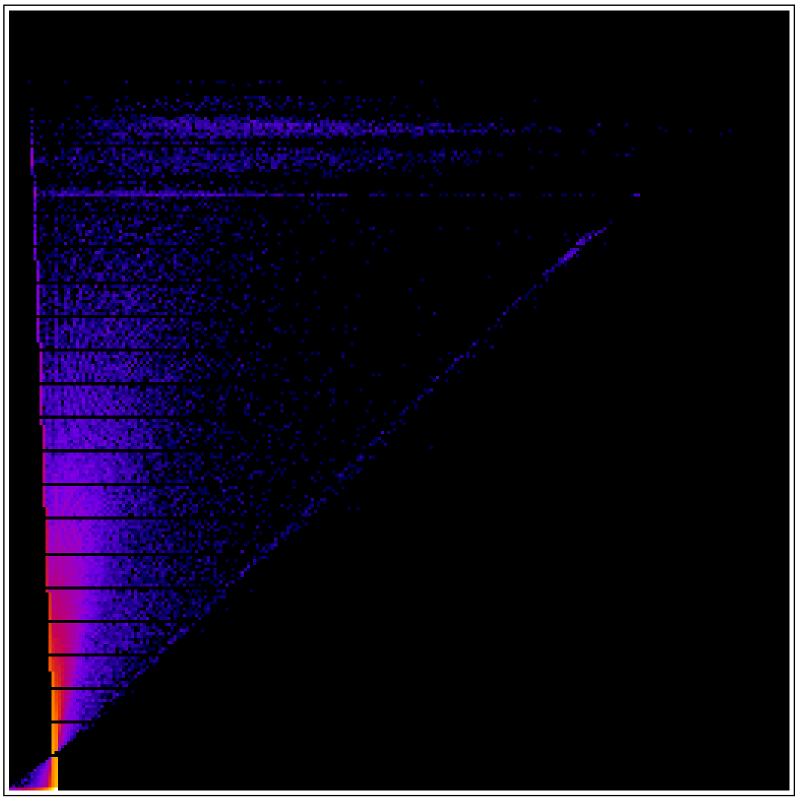

Li - Ch2

256.0 x 256.0

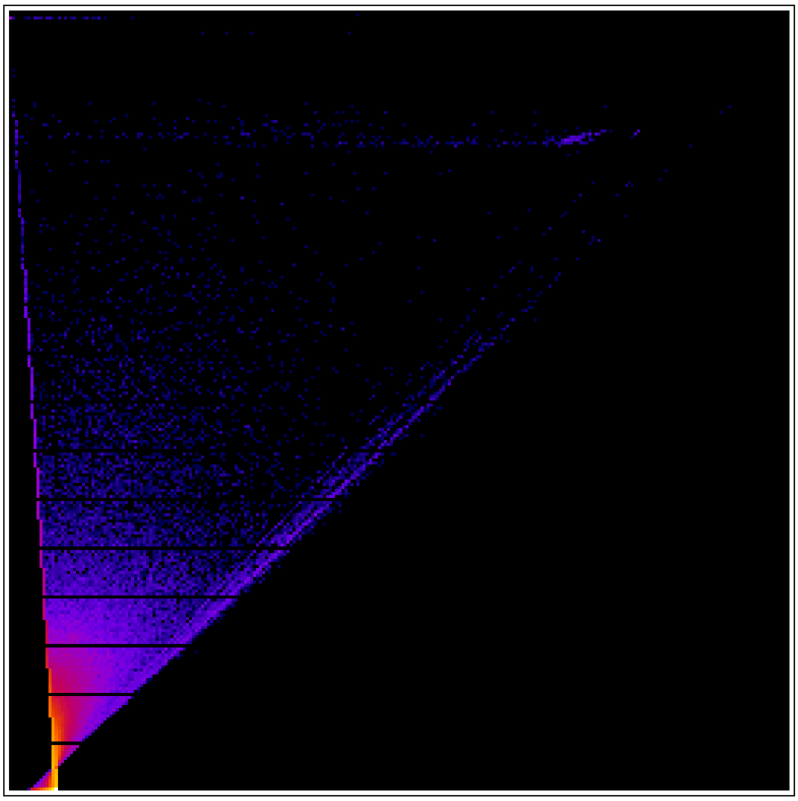

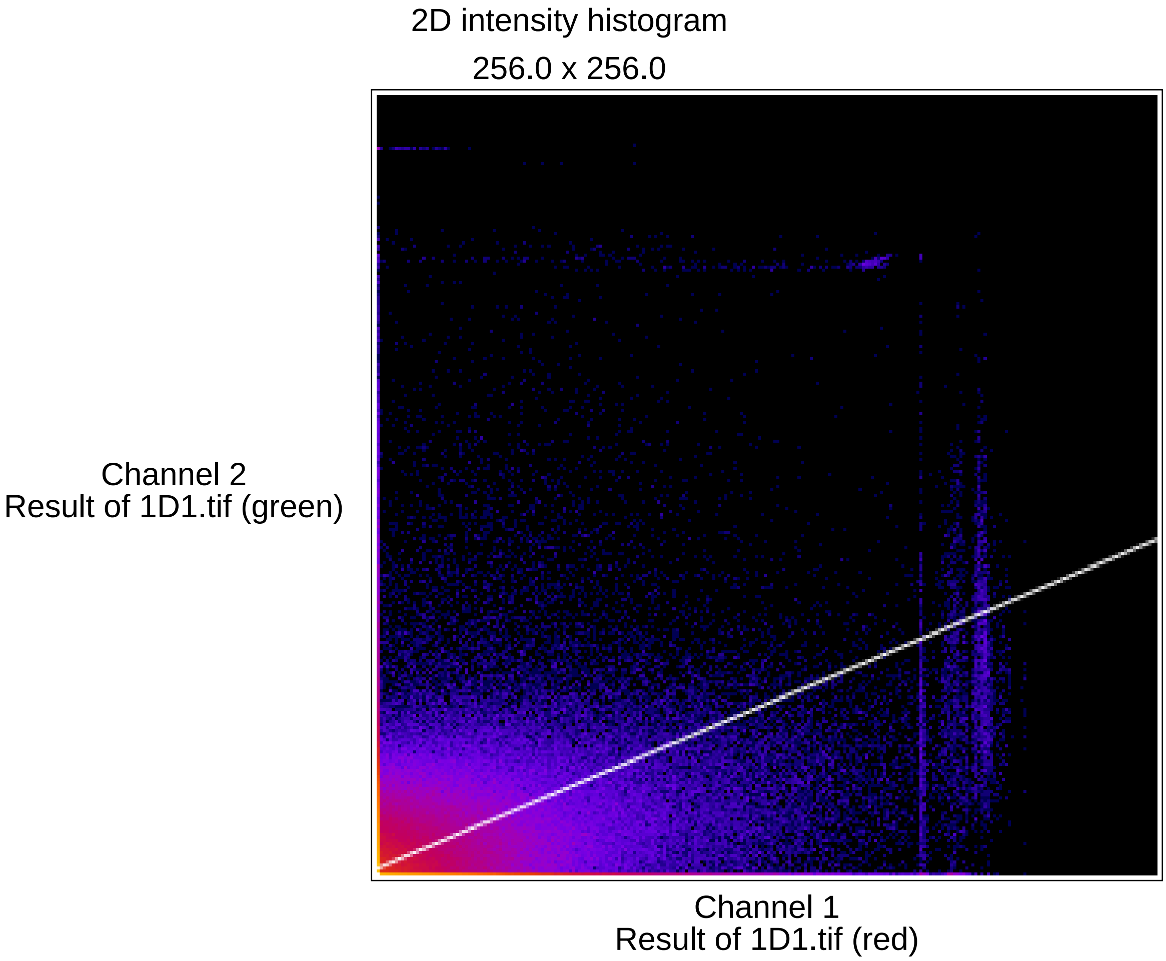

Warning! Zero-zero ratio too high - The ratio between zero-zero pixels and other pixels is large: 0.50. Maybe you should use a ROI.

Warning! y-intercept far from zero - The ratio of the y-intercept of the auto threshold regression line to the mean value of Channel 2 is high. This means the y-intercept is far from zero, implying a significant positive or negative zero offset in the image data intensities. Maybe you should use a ROI. Maybe do a background subtraction in both channels. Make sure you didn't clip off the low intensities to zero. This might not affect Pearson's correlation values very much, but might harm other results.

Warning! Threshold of ch. 1 too high - Too few pixels are taken into account for above-threshold calculations. The threshold is above the channel's mean.

Warning! Threshold of ch. 2 too high - Too few pixels are taken into account for above-threshold calculations. The threshold is above the channel's mean.

Coloc_Job_Name: Colocalization_of_Result of 1D1.tif (red)_versus_Result of 1D1.tif (green)_223345527

% zero-zero pixels: 49.84

% saturated ch1 pixels: 0.00

% saturated ch2 pixels: 0.00

Channel 1 Max: 232.000

Channel 2 Max: 239.000

Channel 1 Min: 0.000

Channel 2 Min: 0.000

Channel 1 Mean: 11.069
Channel 2 Mean: 6.930
Channel 1 Integrated (Sum) Intensity: 11606635.000
Channel 2 Integrated (Sum) Intensity: 7266459.000
Mask Type Used: none
Mask ID Used: 223345527
m (slope): 0.47
b (y-intercept): 1.75
b to y-mean ratio: 0.25
Ch1 Max Threshold: 19.00
Ch2 Max Threshold: 11.00
Threshold regression: Costes
Pearson's R value (no threshold): 0.48
Pearson's R value (below threshold): -0.00
Pearson's R value (above threshold): 0.25
Li's ICQ value: 0.268
Spearman's rank correlation value: 0.37816474
Spearman's correlation t-statistic: 418.3043
t-statistic degrees of freedom: 1048574.000
Manders' M1 (Above zero intensity of Ch2): 0.733
Manders' M2 (Above zero intensity of Ch1): 0.705
Manders' tM1 (Above autothreshold of Ch2): 0.574
Manders' tM2 (Above autothreshold of Ch1): 0.542
Kendall's Tau-b rank correlation value: 0.3248
Costes P-Value: 1.00
Costes Shuffled Mean: 0.00
Costes Shuffled Std.D.: 0.01
Ratio of rand. Pearsons >= actual Pearsons value : 0.00
